# Measurement of Gender Differences of Gastrocnemius Muscle and Tendon Using Sonomyography during Calf Raises: A Pilot Study

**DOI:** 10.1155/2017/6783824

**Published:** 2017-12-31

**Authors:** Guang-Quan Zhou, Yong-Ping Zheng, Ping Zhou

**Affiliations:** ^1^The School of Biological Science & Medical Engineering, Southeast University, Jiangsu, China; ^2^Interdisciplinary Division of Biomedical Engineering, The Hong Kong Polytechnic University, Hung Hom, Hong Kong

## Abstract

Skeletal muscles are essential to the gender-specific characteristics of human movements. Sonomyography, a new signal for quantifying muscle activation, is of great benefit to understand muscle function through monitoring the real-time muscle architectural changes. The purpose of this pilot study was to investigate gender differences in the architectural changes of gastronomies muscle and tendon by using sonomyography during performing two-legged calf raising exercises. A motion analysis system was developed to extract sonomyography from ultrasound images together with kinematic and kinetic measurements. Tiny fascicle length changes among seven male subjects were observed at the initial part of calf raising, whereas the fascicle of seven female subjects shortened immediately. This result suggested that men would generate higher mechanical power output of plantar flexors to regulate their heavier body mass. In addition, the larger regression coefficient between the fascicle length and muscle force for the male subjects implied that higher muscle stiffness for the men was required in demand of maintaining their heavier body economically. The findings from the current study suggested that the body mass might play a factor in the gender difference in structural changes of muscle and tendon during motion. The sonomyography may provide valuable information in the understanding of the gender difference in human movements.

## 1. Introduction 

Skeletal muscles are important human tissues to regulate force generation and control body motions as biological motors [1]. The force generation capabilities of skeletal muscles have been studied for decades to understand the gender-specific characteristics of human movements [2–5]. Several studies reported that men produced greater absolute strength than women [2–4]. The difference was significant even after adjusting for body weight, body mass index (BMI), the cross-sectional area of muscle, or fiber size. Men also had a greater rate of force development compared with women during maximum voluntary contraction (MVC), suggesting their faster force generation abilities [5]. These gender differences can be attributed to many factors, including architectural characteristics of muscle, muscle fiber type, muscle biomechanical characteristics, and neural activity during muscle contractions [6–8].

Muscle architecture, primarily represented by the geometric layout of fascicles within the muscle, such as fascicle length (FL) and pennation angle (PA), is closely related to the muscle function [9, 10]. Muscle imaging has become a promising field of research to recognize the biological and bioelectrical characteristics of muscles through checking the muscle architectural change. As a low cost, widely available, and radiation-free imaging modality, ultrasound imaging has been used in muscle imaging for examining the static changes of FL and PA in response to contraction [10], aging [11], physical training [12], and fatigue [13]. Ultrasound imaging has also been applied to investigate the contribution of muscle architectural difference to gender-specific characteristics in force generation capabilities [14–20]. Males tended to have larger PA in comparison with females [15–17, 19], though the differences in some muscles were not significant [15, 16]. And men compared with women had significantly larger optimal PA for generating maximal force during MVC [20]. Muscles with greater PA allow more fibers to be arranged in parallel within a given cross-sectional area, suggesting a higher force generation potential [18]. These observations can partly explain the difference in force-generating capability between the genders. However, there is a controversy regarding the FL between the sexes in previous literature [14–17]. It is still unclear whether the FL would contribute to the gender-specific characteristics of force generation.

Recently, sonomyography (SMG), representing the real-time change of muscle architecture obtained using ultrasound imaging, was proposed as a noninvasive option for measuring the muscle activation to comprehend the characteristics of human movements in vivo [21]. SMG has been demonstrated to be a very useful and reliable research tool to evaluate how muscle operates during motion [22–26] and diagnosis and rehabilitation assessment in vivo [27–30]. SMG is of great benefit to the understanding of human movements by measuring muscle activation through the real-time muscle architectural changes, though the application of SMG requires extra efforts which demand further considerations of various issues including the synchronization between the ultrasound scanner and the motion analysis system, ultrasound probe placement, and ultrasound data collection and processing. However, there is still a lack of report about using SMG for examining the gender-specific characteristics of muscle architectural change, such as fascicle-shortening range. Moreover, the architectural changes of muscle were facilitated by the tendon elasticity for efficient movement performance [24]. Although previous studies have revealed gender differences in the stiffness of joint, muscle, and tendon [16, 31], the interaction between muscle and tendon tissue has not been investigated using SMG for the gender-specific utilization of tendon elasticity in mechanical demands during movement.

The purpose of this pilot study was to apply SMG to examine the gender-specific architectural changes of the gastrocnemius (GM) muscle and its tendon during performing the two-legged standing calf-raise exercise. The plantar flexor plays an essential role in different functional tasks, such as walking, running, and hopping [22, 32, 33]. The calf-raise exercise has been proven to be capable of accessing the strength and power in the plantar flexors [34]. Also, the muscle activity in the GM and soleus during walking was similar to that in the calf raise [35]. We, therefore, chose to study the standing calf raises exercise to understand the contribution of gender-specific architectural changes of GM muscle to the plantar flexor. In this study, we developed a human motion analysis system with SMG, which consisted of an ultrasound scanner with custom-designed flat probe and software to collect the ultrasound images, a vision-based motion capture system to measure the kinematic and kinetic data, a custom-built device to synchronize the measurements of the ultrasound scanner and motion capture system, and a custom-designed platform to process and analyze the data. We hypothesize that this newly developed motion analysis system could capture and quantity muscle architectural change during motion and that there exist gender-specific differences in the architectural changes of GM muscle and tendon during calf-raise exercises.

## 2. Materials and Methods

### 2.1. Motion Analysis System with SMG

**Figure 1:**
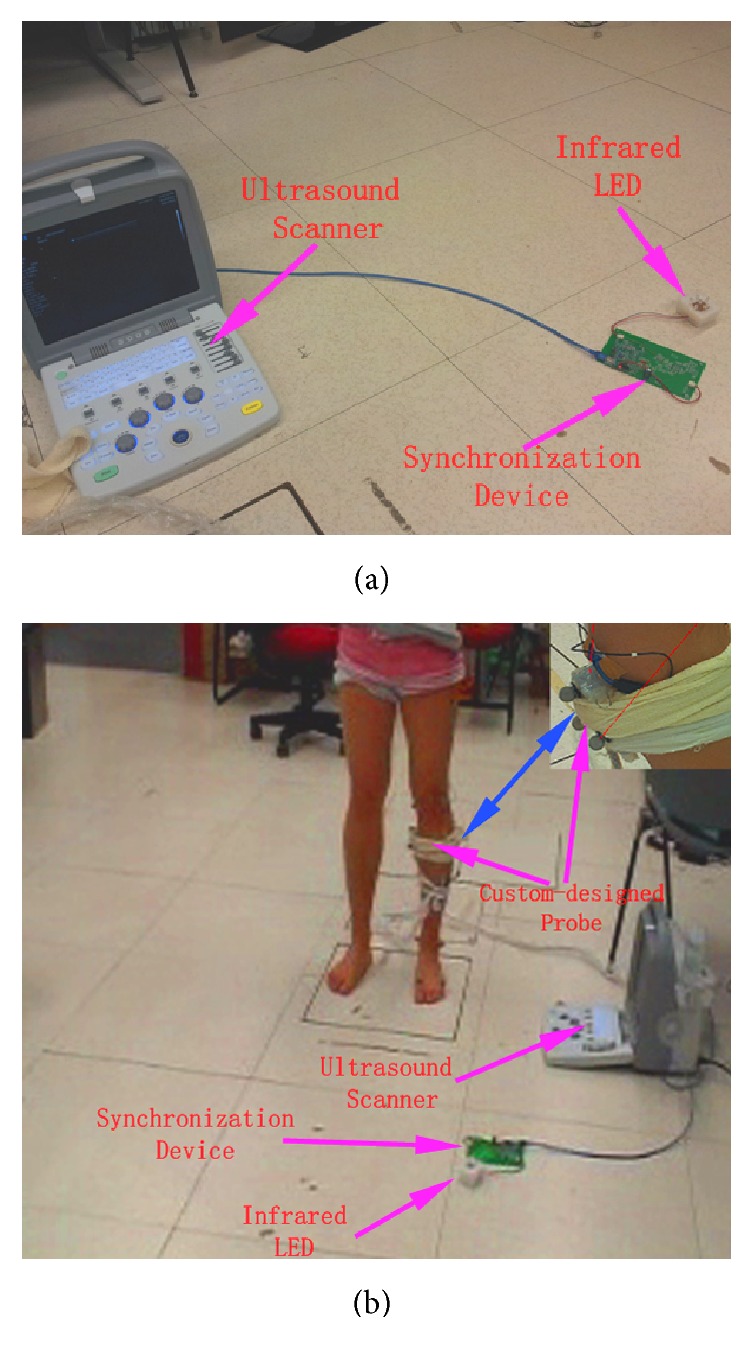
The ultrasound scanner with custom-designed probe and synchronization device: (a) the custom-designed synchronization device; (b) the custom-designed probe was attached on the muscle belly of GM.

The developed motion analysis system mainly consisted of three parts: motion capture system, portable ultrasound instruments with the custom-designed flat probe, and a custom-built synchronization device (Figure 1). In this study, a vision-based motion capture system (VICON MX system, VICON Corp., California, USA) was used for accurate kinematic measurements with eight cameras sampled at 100 Hz. A force sensing platform (ATMI OR6 Series, Advanced Mechanical Technology Inc., Massachusetts, USA) was deployed to measure the gravity reaction force (GRF) for the kinetic analysis. The raw force and moment signal were sampled at 1 kHz and transmitted to the motion capture system. Moreover, the GRF together with the kinematic data was stored in the VICON MX system for further processing. As shown in Figure 1, a portable ultrasound scanner (SIUI1100, Shantou Institute of Ultrasonic Instruments Co., Ltd., Guangdong, China) together with a custom-designed probe of 38 mm in width and 5–10 MHz in frequency was adopted as SMG measurement instrument. The custom-designed flat probe enclosed with silicone was used to make the attachment steadfast and avoid the probe tilt during motion. A custom-built control circuit board with the infrared LED was connected to the ultrasound scanner through universal serial bus (USB) communication (Figure 1), and the infrared LED on the circuit board was used to synchronize the data acquisition of the motion capture system and the ultrasound scanner. Custom-designed software programmed using Visual Studio (Microsoft Corporation, Washington, USA) was integrated on the ultrasound scanner for controlling the synchronization device and capturing ultrasound images directly from the ultrasound scanner. During the data acquisition, the infrared LED on the circuit was switched on by the custom-designed software, which can be recognized as the external synchronization signal to the motion capture system. Simultaneously, the ultrasound images were directly stored on the ultrasound scanner (21 frames/s), which avoided the additional time-delay and possible artifacts if using extra video capture card. The time index of each image was recorded for better alignment between the measurements of motion capture system and ultrasound scanner.

**Figure 2:**
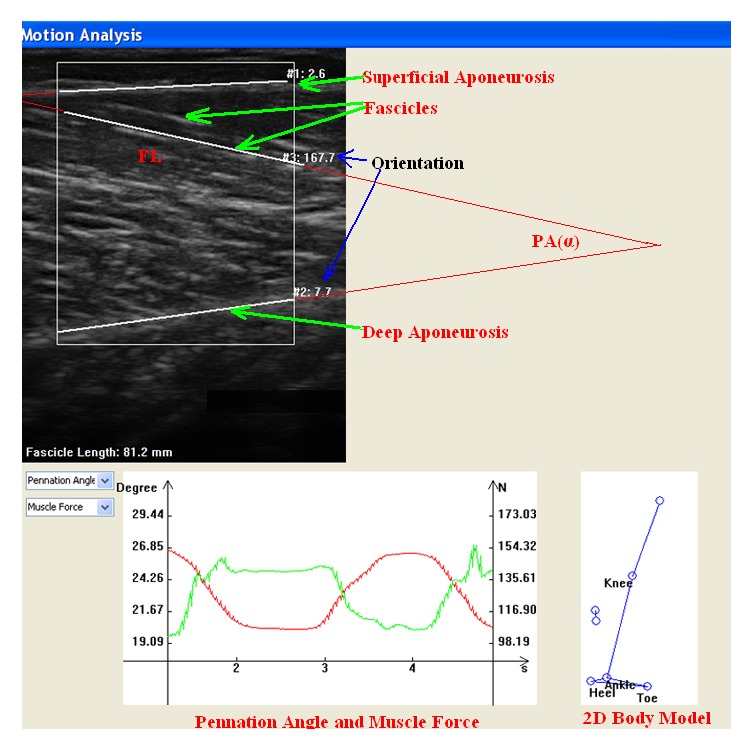
The FL and PA as defined in GM ultrasound image on the interface of custom-designed data analysis platform.

After the data acquisition, the data stored in the motion capture system was picked out to synchronize with ultrasound data by using the signal indicated by the infrared LED. And the selected data together with ultrasound images was then exported into the custom-designed data analysis program developed with Visual Studio (Microsoft Corporation, Washington, USA) and visualization tool OpenGL for data processing and analysis. The kinematic analysis, such as joint angle change, was achieved through calculating the spatial data of the human body, while the SMG, including FL and PA (Figure 2), was automatically derived from the ultrasound images. Some automated approaches had been developed to extract the SMG from ultrasound images [25, 26, 36–40]. In the current system, the automatic extraction of SMG was achieved by applying a number of our previously developed image processing techniques [25, 26, 39, 40]. Moreover, the GRF with kinematic data and SMG could be used to calculate tendon and muscle force through inverse dynamics [41], thus allowing the estimation of muscle force-length relationship.

### 2.2. Experimental Protocol

Fourteen healthy normal subjects (seven male and seven female) with no history of musculoskeletal injury were recruited from the authors' institute and participated in the study. Exclusion criteria were musculoskeletal disorders in the lower limb that prevented participation in typical activities greater than 1 day, current pain in the lower limb or trunk, or being engaged in some regular exercise (physical activity at least 3 times per week). The mean body weight and BMI of the subjects were 52.3 (6.4) kg and 20.1 (2.7) kg/m^2^ for the women (age: 29.0 (4.0) years) and 77.2 (10.5) kg and 24.8 (3.2) kg/m^2^ for the men (age: 34.7 (6.8) years). The institutional ethical committee approved this study, and all subjects gave written informed consent prior to participation in the experiment.

The reflective markers were attached to the subject's left leg with medical proof fabric on the basis of the uOttawa marker set because this enhanced version of the Plug-in-Gait marker set could increase the reproducibility of joint kinematics and kinetics during motion [42]. These reflective markers were placed over the lower lateral surface of the thigh, the lateral and medial epicondyle of the knee, the lower shank, the lateral and medial malleolus, heel, and the first and fifth metatarsal head, respectively. As the measurements along the mid-sagittal axis were found to be representative for the fascicle measurements both at rest and in the contracted state [10], the custom-designed flat probe was secured steadfastly on the mid-belly of GM with the bandage. The orientation of probe was carefully adjusted to visualize the fascicles from deep to superficial aponeuroses. Enough ultrasound gel was also applied on the region of measurement to fill the gap between the probe and the skin so as to reduce the artificial effect in ultrasound images caused by motion. Before data acquisition, the subjects were instructed to perform the two-legged calf raises freely in the laboratory to facilitate adaptation to the speed, heel height, and the laboratory environment. During data collection, the subject stood on the force platform and repeatedly raised up on their tiptoes with their body in an upright posture. The subjects were instructed to rise onto their toes as high as possible and followed a rhythm of 1 Hz produced with an electronic metronome. The subjects raised and lowered their heels each for approximately 1 second, which was similar to the previous studies [35, 43]. The examination was repeated three times with a rest of 1 minute between two consequent trials, and each trial lasted for about 20~30 seconds.

### 2.3. Data Analysis

The architectural changes in the muscle and tendon were analyzed together with the ankle plantar-flexion angle. The plantar-flexion angles of the ankle were calculated by the obtained spatial data of the reflective markers. The length of GM muscle-tendon unit (MTU), *L*_MTU_, was estimated from the joint angular data and the shank length with the model of Hawkins and Hull [44]. As shown in Figure 2, PA was defined as the angle of fascicle to deep aponeurosis, and FL was defined as the length along the fascicular path from the superficial to deep aponeuroses. In cases in which the fascicle extended off the ultrasound image, the length of the fascicle was estimated by extrapolating both the visible path of fascicle and the aponeuroses in the image linearly. According to the definitions, the PA (*α*) and FL (*L*_Fas_) of GM were automatically extracted from the B-Mode images by applying a number of image processing techniques [25, 26, 39, 40]. The FL and PA obtained with the automatic methods were visually examined for guaranteeing the measurements of muscle architecture. Finally, the Achilles tendon length was calculated by using the MTU length (*L*_MTU_), the FL (*L*_Fas_), and the PA (*α*):

(1) $$\begin{array}{c} L_{Ach}=L_{MTU}-L_{mus}=L_{MTU}-L_{Fas}\cdot \cos\left(\;\alpha \;\right). \end{array}$$

The joint torque can be calculated with the GRF and kinematic data through inverse dynamics [41]. Because the moment arm length of Achilles tendon, *M*_A_, has been reported to be a function of ankle joint angles [45], the Achilles tendon force was estimated from the ankle joint torque *T*_Ankle_ and the moment arm length of Achilles tendon:

(2) $$\begin{array}{c} F_{Ten}=\frac{T_{Ankle}}{M_{A}}. \end{array}$$

The relative contribution of force developed by the GM to the Achilles tendon force was assumed to be equal to the relative physiological cross-sectional area (PCSA) of the GM within all plantar flexors [23]. The GM PCSA takes up 15.4% of the total PCSA among all plantar flexors [32]. The GM muscle force can then be calculated with

(3) $$\begin{array}{c} F_{GM}=\frac{\left(0.154*F_{Ten}\right)}{\cos\left(\alpha \right)}. \end{array}$$

The regression coefficient (slope) of the muscle force-length was then calculated as the recruited muscle stiffness in calf raises. Moreover, as the range of plantar-flexion ankle angle in humans has significant variations in the activities of daily life [46, 47] and the ankle plantar-flexion range is usually below 25° during walking [48, 49], the changes of muscle and tendon within 25° plantar-flexion ankle angle were examined between the male and female subjects.

### 2.4. Statistical Analysis

Values were presented as mean (SD). One-way analysis of variance (ANOVA) was used to test the effect of gender on the height, body mass, BMI, muscle force, tendon force, FL, PA, and tendon length, respectively. The linear regression analysis was employed to describe the relationship between the FL and muscle force and between the tendon length and tendon force. Pearson product-moment correlations (*r*) were calculated to measure the correlation. Student's paired *t*-test was applied to check the differences between the male and female groups for the changes in FL, PA, and tendon length. Moreover, the regression coefficient (slope) and area of force-length relationships between the male and female subjects were examined with Student's paired *t*-test. The linear regressions analysis was also implemented to verify the relationship between the slope and body mass for both genders. In each test, the level of significance was accepted if *P* < 0.05. As well, Cohen's *d* effect size was calculated as an indicator of the magnitude of the difference between groups, considering large effect sizes as clinically relevant differences (i.e., |*d* | >0.8) [50].

## 3. Results

During calf-raise exercises, the FL ranged from 74.7 (6.7) mm to 35.0 (6.7) mm, while the range of PA was from 18.3 (2.8)° to 36.8 (6.4)°. And the average difference in tendon length was 26.1 mm. The FL of the females and males was 73.0 (5.3) mm and 76.4 (7.9) mm at rest (Cohen's *d* = 0.51), while the PA was 17.4 (2.2)° and 19.2 (3.2)° (Cohen's *d* = 0.66), respectively. Although the PA and length of fascicle and tendon were larger in males, these gender differences were not significant (all *P* > 0.05). In contrast, compared with the female subjects, the peak plantar flexor moment and force of their counterparts were significantly larger (torque: male = 48.6 (8.6) Nm, female = 30.6 (5.5) Nm, Cohen's *d* = 2.5; force: male = 911.1 (163.9) N, female = 581.5 (111) N, Cohen's *d* = 2.35; all *P* < 0.001).

**Figure 3:**
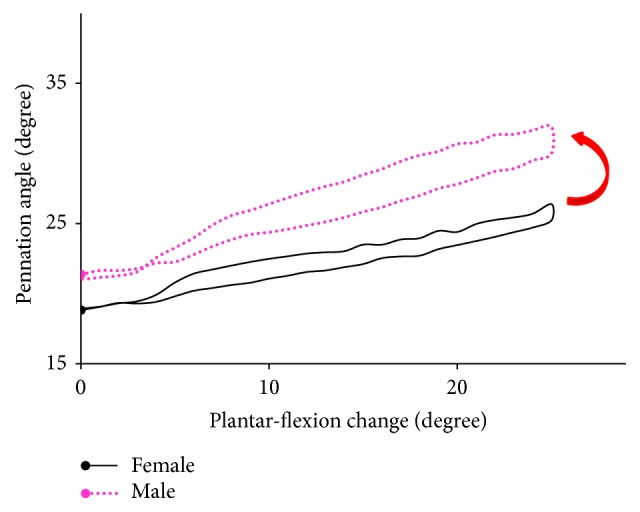
The average pennation angle change for seven male subjects and seven female subjects within the plantar-flexion angle change of 25°. The arrow in red indicates the direction of raising and lowering subjects' heels.

**Figure 4:**
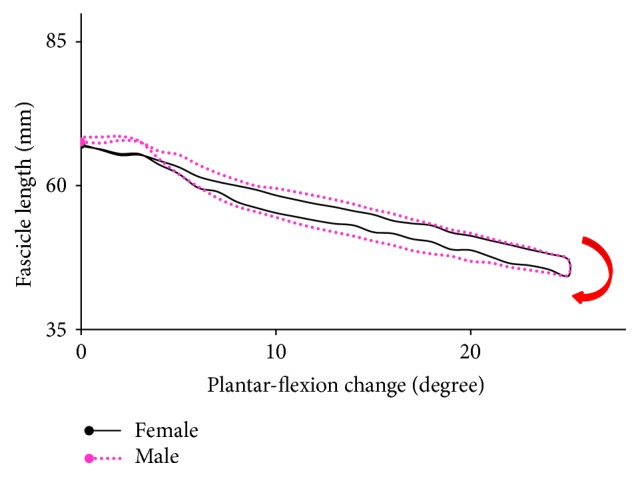
The average fascicle length change for seven male subjects and seven female subjects within the plantar-flexion angle change of 25°. The arrow in red indicates the direction of raising and lowering subjects' heels.

The length change of fascicle and tendon was almost the same between the genders when the plantar-flexion angle was changed by 25° (Table 1), while the change of PA for the men was 3° larger (*P* = 0.06, Cohen's *d* = 1.12). As shown in Figure 3, the difference in PA between the males and females became larger with the change of plantar-flexion angle. This suggests that more fibers would be arranged in parallel within a given CSA for men when performing the calf raises exercises. The pattern of fascicle change was also observed to be different, particularly in the initial part of calf raises, though the range of FL was almost the same between the genders (Figure 4). The FL of men was kept nearly constant at the initial part of calf raises, while the fascicle of women shortened by 2.3 mm. This would contribute to the significant difference in peak plantar flexor moment and force that was required to support their different body mass for both genders (all *P* < 0.001).

**Figure 5:**
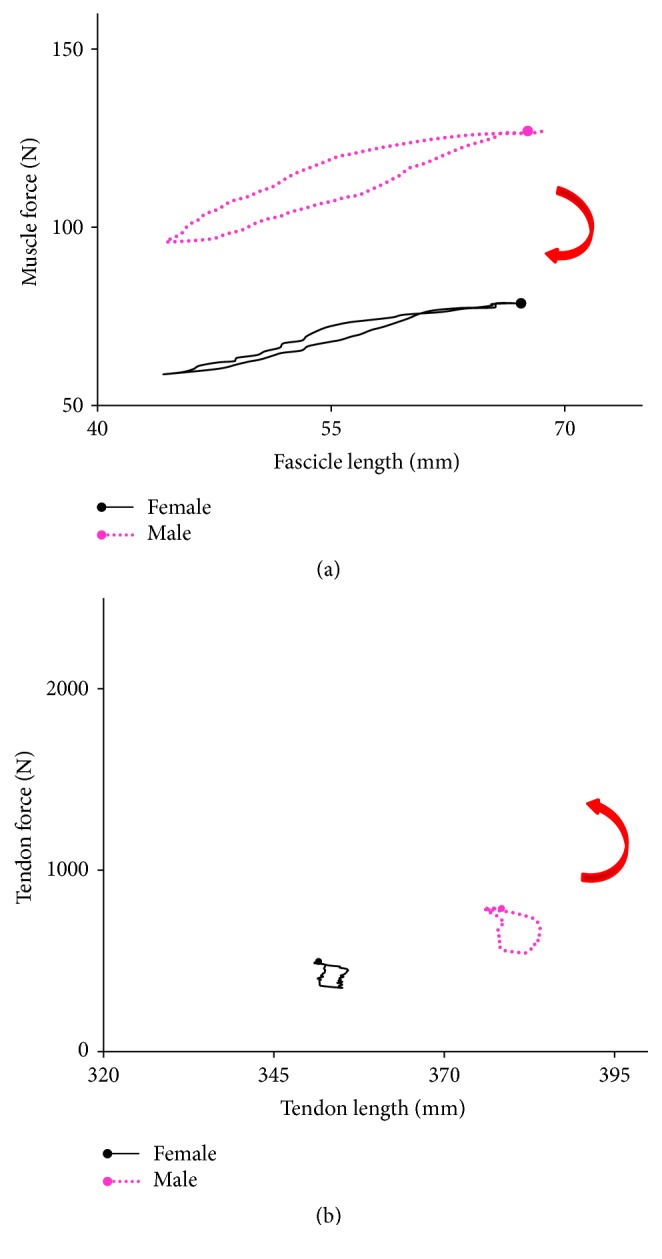
The force-length relationships of muscle and tendon. Average of seven male subjects and seven female subjects within the plantar-flexion angle change of 25°: (a) the relationship between muscle force and fascicle length; (b) the relationship between tendon force and tendon length. The arrows in red indicate the direction of raising and lowering subjects' heels.

**Figure 6:**
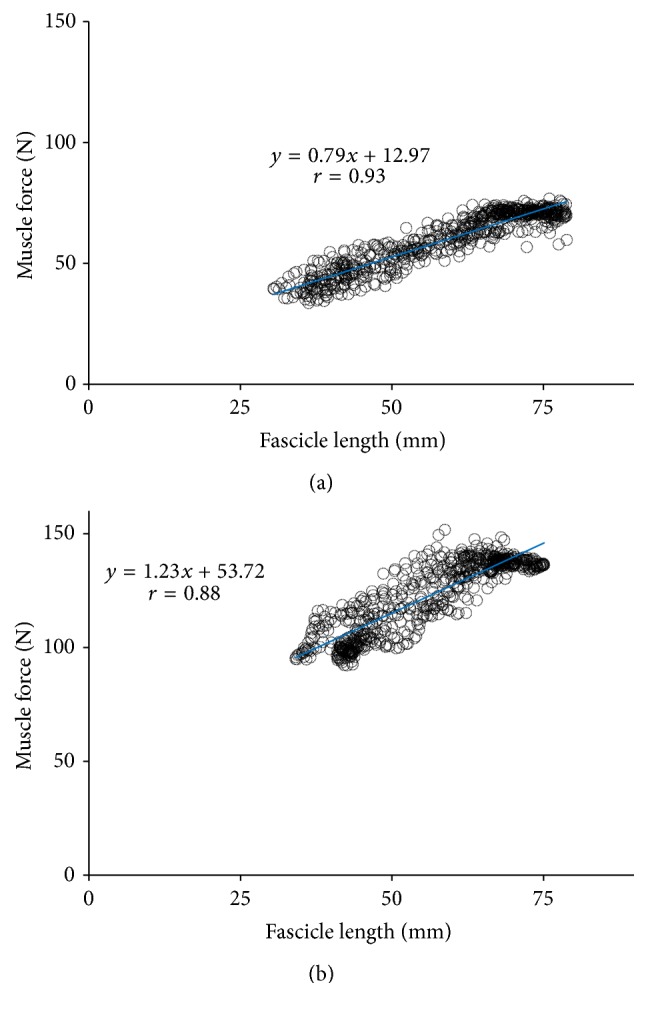
The cross-correlation between fascicle length and muscle force during calf raises: (a) the cross-correlation between fascicle length and muscle force for one typical female subject; (b) the cross-correlation between fascicle length and muscle force for one typical male subject.

Figure 5 shows the force-length relationships of the GM muscle and tendon within the plantar-flexion angle change of 25° for both the males and females. The areas made by force-length relationships were significantly larger for men for both the muscle and tendon (muscle: *P* = 0.041, Cohen's *d* = 1.22, Figure 5(a); tendon: *P* = 0.049, Cohen's *d* = 1.17, Figure 5(b)). This implied that more mechanical energy was generated and dissipated by men when performing the calf raises exercises. The tendon length was not significantly linearly correlated with the tendon force during the plantar-flexion exercises (*r* = 0.37 (0.19)). On the other hand, the linear correlation between the FL and muscle force was significant for both the genders (male: *r* = 0.87 (0.04), all *P* < 0.001; female: *r* = 0.86 (0.07), all *P* < 0.001).  Figure 6 shows the typical correlation between the FL and muscle force for one male and one female subject during experiments. Moreover, although the difference in the slope of the relationship was not significant (*P* = 0.12), the regression coefficient (slope) of the males (slope = 1.28 (0.34) N/mm) was larger than that of the females (slope = 0.96 (0.37) N/mm). Also, large effect size (Cohen's *d* = 0.90) was found for the regression coefficient. After normalizing to the body weight, there is almost no difference in the slope between the genders (male: normalized slope = 0.017 (0.04); female: normalized slope = 0.018 (0.05); *P* = 0.62, Cohen's *d* = 0.02). Furthermore, the slope was highly correlated with their body mass for the females (*r* = 0.90), whereas there is a weak linear correlation between the body weight and slope for the males (*r* = 0.38). When only using the slope of the male subjects with BMI less than 25 kg/m^2^, the linear correlation became more correlated (*r* = 0.82).

## 4. Discussion

We have investigated the effect of gender on the architectural changes of GM muscle and tendon undergoing the cyclic two-legged calf raises exercises. The main finding of this study was that the pattern of GM fascicle change was different at the initial part of calf raises between the men and women (Figure 3), though no significant difference in the length change of GM fascicle and tendon was observed between them. It has been reported that body weight and physique emerged as a significant predictor of muscle strength of the ankle plantar flexors between the genders [3, 51] and might be affecting the gait pattern [52, 53] and muscle endurance [54]. Since the male participants in this study were approximately 25 kg heavier than the female participants, the difference in GM fascicle change can be partly attributed to the body weight difference between the genders. In addition, a greater regression coefficient between the FL and muscle force (female: 0.96 (0.37) N/mm; male: 1.28 (0.34) N/mm) was observed for the males in calf raises, implying that higher muscle stiffness for the men was recruited for supporting the body weight economically.

The changes of fascicles were observed to be different between the genders (Figure 3). The fascicle of the female subjects shortened immediately at the initial part of calf raises, while that of the male subjects kept nearly constant. As both the raising and lowering portions took approximately 1 second during calf raises, this result inferred that shortening velocity of the males was slower than that of the females at the initial part of calf raises under the same ankle angular speed. A lower shortening velocity would be beneficial for the mechanical power output at the initial part of calf raises because it operated around the plateau region of the force-length curve. According to a previous study in gait analysis, fascicle shortening at the time of peak force production was shifted to much slower velocities when switching from a walking gait to a running gait at the same speed, resulting in a substantial increase in peak muscle force and an increase in GM power output [22]. In the squat jump, the countermovement jump, and drop jump, the fascicles also worked in the relatively low-shortening velocity region, especially in the late push-off phase, which enables the fascicles to generate relatively high force according to the force-velocity relationship [23, 24]. Our findings together with the previous results suggest that the GM muscle of men produces high forces at a relatively optimal level with small amounts of work and high efficiency at the initial part of calf raises. Since the body weight and fat-free mass have been reported to be one of the important contributors to jump performance and strength at all lower limb joints [3, 15], this difference in fascicle changes might be attributed to the heavier body mass of men. The acceleration of the leg into the swing and forward kinetic energy of the trunk were mainly dependent on the ankle plantar flexor work in the stance phase of walking [22, 33]. And an increase of vertical velocity of the mass center of the body during the late push-off phase required a high mechanical power output of plantar flexors [23]. Therefore, the little change of FL at the initial part of calf raises would enable the muscle to operate near its highest force region, serving to generate higher mechanical power output of plantar flexors to support the heavier body weight of men.

The fascicle and tendon exhibited the viscosity behavior during the repeated two-legged calf-raise exercises in the present study (Figure 5). The MTU exhibited viscoelastic properties for efficiently utilizing the outcome of muscle contraction during human movement [24, 55], which can be ascribed to the elasticity of cross-bridge, the actin, and possibly the myosin filaments. The viscoelastic characteristics of MTU play a significant role in stretch-shortening cycles in which an eccentric action is immediately followed by a concentric action of working muscles. Tendon acted as an energy storing spring by springing in the cyclic plantar flexion, to contribute a considerable amount of energy to the total mechanical work performed [23]. The area within the tendon force-length curve, that is, hysteresis, was the energy loss mainly due to converting mechanical work into heat, while the area under the descending limb of the force-length curve represented the energy recovered from tendon [56, 57]. Although higher mechanical power output for the men (Figure 5(a), *P* = 0.041) was generated during calf-raise exercises, the significantly larger hysteresis area of the tendon force-length curve in the male subjects that indicated more energy was dissipated in the cyclic calf raises exercises (Figure 5(b), *P* = 0.049). This observation of greater hysteresis in males was in agreement with the finding of a previous investigation reported by Kubo et al. [56]. This can partly explain the muscle endurance difference between the genders when performing stretch-shortening cycle exercise [5, 51].

The tendon was elongated slightly under the lower Achilles tendon force. When tendons were forcibly stretched, they responded in a nonlinear fashion, with an initial curvilinear toe region followed by an approximately linear region [58]. The elongation of the tendon increased in a curvilinear pattern, implying that in vivo tendons may exhibit creep [59]. During walking, the elongation of triceps surae elastic structures can reach up to 4%, which operates in the toe region of tendons [1]. An interesting finding of this study was that no significant linear correlation was observed between the tendon force and tendon length, which may suggest that the tendons operated in the curvilinear toe region during calf-raise exercises. On the other hand, the muscle force was significantly correlated with the FL. The regression coefficient (slope) of the muscle force-length relationship for the men compared with the women was larger, while the slope of the men became almost the same as that of the women after normalized to the body weight. Some previous studies showed that both the passive and active muscle stiffness in women was lower than that in men [6, 60, 61]. Muscle stiffness difference between genders was likely to be attributed to a gender difference in the viscoelastic properties of the muscle [61]. It has been reported that the muscular system of males was more efficient in resisting changes in its length [6], implying stiffer muscle involved in contraction. Therefore, the larger slope of the male subjects in this study may suggest stiffer muscle recruited in calf raises. Moreover, muscle mass and body weight could further explain the majority of the gender effect in leg stiffness and muscle stiffness [16, 31, 60, 62]. Male subjects recruited higher leg stiffness to drive their heavier body mass than the lighter female subjects during performing functional tasks [31, 62]. As active muscle stiffness contributed to leg stiffness [31], the larger slope observed in the male subjects may imply that males recruited stiffer muscle to support the heavier body weight of men economically in calf raises. Body mass was also an important contributor to the between-subject difference in stiffness [16], which can explain the strong correlation between the body weight and slope for the females (*r* = 0.90) in the present study. However, the slope was not highly correlated with the body mass for the males (*r* = 0.38). Body fat has been demonstrated to be significantly positively correlated with BMI [63, 64]. The BMI of 25 kg/m^2^ in men and 23 kg/m^2^ in women has been suggested as diagnostic screening cut-offs for obesity [64]. Thus, the observed nonsignificant correlation between the body weight and slope might be partly attributed to the higher BMI in the males in this study (male: 24.8 (3.2) kg/m^2^; female: 20.1 (2.7) kg/m^2^). When only using the slopes for the male subject with BMI less than 25 kg/m^2^, the correlation became more correlated (*r* = 0.82). Further study is required to explore the influence of body mass and body fat in more detail by recruiting more subjects.

The changes and values of FL and PA measured with the proposed method were consistent with the previous reports [9, 10, 58, 65]. In this experiment, the mean FL at rest was 74.7 mm and was reduced to 35.0 mm during calf raises, while the range of average PA was from 18.3° to 36.8°. The average length of GM fascicle for healthy subjects was 78 mm as the knee was fully extended and the ankle reached 15° dorsiflexions [65]. With the knee fully extended and the ankle fixed at its neutral position, the fascicles reportedly shortened to 30~34 mm during MVC, while the PA increased to 35~40° [58]. Both the PA and FL in the males were found to be larger than those of the females in this study, though the differences were not significant (*P* = 0.23 and *P* = 0.37, resp.). Greater PA in males is well agreed in previous literature [15–17, 19, 20]. However, there are contradictive results regarding the FL in different genders [14, 15, 17, 19]. Alegre et al. [15] reported longer fascicles of GM muscle in men. In contrast, women were reported to have larger FL in GM muscle [14, 17]. Previous studies found that training can induce the necessary adaptation of fascicle geometry [12, 66]. The gender-specific difference in GM fascicle depended on the training or regular exercise mode [19]. The habit of routine exercise might contribute to the variance in FL in both genders. Thus, the gender might not be the primary determinant of GM fascicle length. In future, more subjects should be recruited to validate this assumption.

It should be noted in this study that the slope of the force-length relationship was not the actual muscle stiffness. The torque that was subtracted by the torque of relaxed conditions was used to estimate the active muscle stiffness [67]. Thus, the regression coefficient (slope) in the present study only indicated the average muscle stiffness that was the sum of passive and active muscle stiffness recruited in calf raises. However, this factor did not affect the main results of this study. Moreover, there may be fascicle curvature and the heterogeneity of changes in FL within the muscle. The fascicles were identified from the mid-belly of the GM muscles in the present study. Since fascicles are almost straight at rest and become slightly curved with the increase in contraction level and decrease in muscle length, resulting in only ~6% underestimation in FL during maximum voluntary contraction (MVC) [68], which is thus a minor factor on the measurement of FL in the calf raises exercise. The FL was almost uniform throughout the muscle at both rest and submaximal isometric contractions [69]. Narici et al. [10] also reported that the measurements along the mid-sagittal axis were representative for the measurements of fascicles at both relaxed and maximal isometric contracted conditions. Therefore, we believe that the measured fascicles in this study could represent the changes of all fascicles in the GM muscle.

There are still several limitations in this study. Firstly, the influence of physical activity was not well taken into consideration in the present study. All subjects recruited in this study are only required not to exercise regularly or receive any specific physical training. Different training modes have been proven to induce the various adaptation of fascicle geometry [19] and might be affecting behaviors of fascicles and the strength of the ankle plantar flexor during calf raises. The effect of physical activity should be further investigated in future studies by using physical activity score and a cut-off score to clearly establish both groups as equally physically active in the experiment. Another limitation of this pilot study was that our measurements were restricted to the GM muscle, which is just one of the calf muscles responsible for plantar flexor. Also, this study has a relatively small number of participants. Future studies, therefore, should be conducted with a larger sample in each group of sex, other calf muscles, and other movements involving plantar flexion, such as walking and hopping, allowing the better understanding of gender-specific characteristics in plantar flexor muscles.

In conclusion, sonomyography can examine the dynamic geometric properties of muscle and tendon, providing valuable information in the understanding of the gender-specific characteristics during motion. The results of this pilot study indicate that the architectural changes of GM muscle for males allowed their muscles to generate a higher mechanical power output during calf-raise exercises. Moreover, the muscle stiffness of the males recruited in the calf raises exercises was larger than that of the females. These findings suggest that the muscle of men might operate to provide higher output to support their heavier body weight economically. The body mass might be one of the factors in the behavior difference of muscle and tendon between gender. Future research is necessary to determine the influence of body weight on muscle activity during normal gait and their clinical/physiological implications for joint stability and muscle and tendon injury risk.

## Figures and Tables

**Table 1:** The architectural changes of muscle and tendon within the plantar-flexion ankle angle change of 25°.

	Female	Male	Total
Fascicle length (mm)			
Change (*P* = 0.51, *Cohen's* *d* = 0.36)	24.2 (4.1)	25.4 (2.3)	24.8 (3.2)
Pennation angle (°)			
Change (*P* = 0.06, *Cohen's* *d* = 1.12)	8.0 (2.2)	11.2 (3.4)	9.6 (3.2)
Tendon length (mm)			
Change (*P* = 0.39, *Cohen's* *d* = 0.19)	10.7 (3.3)	11.2 (1.7)	10.9 (2.5)
